# Optimizing Patient Information Material for a New Psycho-Oncological Care Program Using a Participatory Health Research Approach in Germany

**DOI:** 10.3390/ijerph19031518

**Published:** 2022-01-28

**Authors:** Theresia Krieger, Sandra Salm, Antje Dresen, Anna Arning, Kathrin Schwickerath, Andrea Göttel, Stefanie Houwaart, Holger Pfaff, Natalia Cecon

**Affiliations:** 1 Institute of Medical Sociology, Health Services Research and Rehabilitation Science (IMVR), University of Cologne, 50933 Cologne, Germany; sandra.salm@uk-koeln.de (S.S.); antje.dresen@uk-koeln.de (A.D.); holger.pfaff@uk-koeln.de (H.P.); natalia.cecon@uk-koeln.de (N.C.); 2 German Cancer Society North-Rhine Westphalia (KG-NRW), 40221 Düsseldorf, Germany; u-a.arning@web.de (A.A.); schwickerath@krebsgesellschaft-nrw.de (K.S.); goettel@krebsgesellschaft-nrw.de (A.G.); 3 House of the Cancer Patient Support Associations of Germany (HKSH-BV), 53111 Bonn, Germany; stefanie.houwaart@posteo.de

**Keywords:** cancer, psycho-oncological support, patient information material, quality assessment, optimization process, participatory health research, patient engagement, participation

## Abstract

High-quality patient information material (PIM) is essential for patients´ informed decision-making, and its quality may influence a care program’s acceptance. In the new psycho-oncological care program, isPO, the initial PIM was developed top-down and required optimization. In this paper, we report on the process and experiences of optimizing PIM’s quality bottom-up by applying a Participatory Health Research (PHR) approach. Cancer-patient representatives of the national peer-support group contributed as co-researchers as part of the optimization team. A mixed-methods design was chosen. First, the quality of the initially utilized PIM was assessed with the newly designed user-friendly instrument UPIM-Check. Next, three Participatory Action Research loops were conducted, including cancers survivors and isPO service providers. The initial isPO PIM’s were assed to be of low quality, limited usability and incomplete. Bottom-up generated optimization suggestions led to the improvement of two initially used PIMs (leaflet, patient information folder) and the design of two new PIMs (poster, study information overview). The optimized PIM facilitates isPO service providers’ care provision and helps newly diagnosed cancer patients in understanding and accepting the new program. PIM optimization benefited from applying PHR. The patient representatives’ contribution and active patient engagement were central for quality assessment and designing needs-driven, mature and complete PIM.

## 1. Introduction

### 1.1. Background

Worldwide, patient information material (PIM) is essential in healthcare [1]. Well-designed high-quality PIM empowers the end-user (e.g., cancer patients) [1]; increases their satisfaction concerning communication with health professionals, especially in complex situations (e.g., with life threatening events) [2]; and augments the openness and motivation to participate in new interventions or research programs [3,4]. PIM can enhance end-users’ program understanding [5] and, therefore, may increase program acceptance [6]. Low-quality PIM may provoke uncertainty, misinterpretation, or patients’ resistance to a program [7,8]. In cancer-care support, PIM’s quality impacts on patients’ anxiety levels, emotional distress, vulnerability, and unfamiliarity with the new situation [9,10]. 

Different materials (e.g., leaflets or posters) are utilized for different activities and purposes in the healthcare system [11]. High-quality PIM should target two objectives: (1) end-user friendly information transmission and (2) a recommendation for action [7]. PIM should be in a readable and motivational style (e.g., use of positive language) and is considered as most effective if it is perceived as being user-friendly [12,13]. Four PIM quality criteria are considered essential: (1) the correctness and validity of content, (2) the readability in respect to text structure and graphic, (3) the comprehensibility for the end-users, and (4) their utility in the field [7,8,14]. International and national checklists, such as Discern^´^ [15] or TEMPtEd [16], aim to support the design and optimization of PIM and its quality assessment [8]. One already existing checklist, the PEMAT [13], helps professionals in assessing PIM´s understandability and actionability. Unfortunately, the available assessment instruments were developed with, and for, experts and researchers, whereas end-users (patients) were rarely engaged. Moreover, most instruments appear to be unsuitable for end-users’ application, due to the required literacy level. Consequently, end-users are hardly involved in PIM assessment [4,17,18]. 

When designing new PIM, end-users are rarely engaged [13,18]. As a result, many materials are of low efficacy, as they contain too much information, include difficult words, or lack recommendations for action [1,17,19,20]. Before utilizing a new PIM, it is recommended to assess its readability and suitability for end-users [1,11]. However, for various reasons, e.g., lack of availability or time, such assessments are rarely conducted [19]. 

The assessment, optimization, and development of PIM can benefit from key stakeholders´ (e.g., end-users or service providers) active participation [18,21]. The PHR approach aims to actively build on stakeholders’ implicit knowledge and experiences by their continuous involvement and engagement [22,23,24,25]. PHR acknowledges the insider perspective of people living with the health problem (e.g., cancer) as a practical and intuitive source [25]. 

In order to enable the co-creation of new knowledge, the empowerment and capacity building of the co-researchers is vital [26]. 

### 1.2. The Integrated Cross-Sectoral Psycho-Oncological (isPO) Program

In Germany, between 2017 and 2022, the Integrated Cross-Sectoral Psycho-Oncological (isPO) care program was recently designed, implemented, and evaluated; it is financed by the federal innovation fund [27,28]. The 12-month psychosocial and psychotherapeutic isPO program is offered to newly diagnosed adult cancer patients, parallel to their biomedical therapeutic treatment [28]. It aims to impact (1) on an individual-patient level, reducing patients’ depression and anxiety due to the cancer diagnosis; and, (2) on a system level, it seeks to offer a high-quality needs-driven psycho-oncological care program for comprehensive implementation into nationwide cancer care [27]. Since January 2019, isPO has been available free of charge for its target group (newly diagnosed cancer patients) in four especially established care networks in North-Rhine Westphalia, Germany. Due to its stepped care approach, as well as the inclusion of several service providers and different components, isPO is considered to be a complex intervention [29]. Complex interventions work best if they are well understood by their end-users and/or service providers. Consequently, the availability of high-quality PIMs seems to be vital for isPO to achieve its purpose [3,4]. 

The Institute of Medical Sociology, Health Services Research and Rehabilitation Science (IMVR) at the University of Cologne conducts external formative and summative program evaluation [27]. Moreover, isPO is currently implemented as part of a project and therefore includes both a program (treatment) component and a study (research) component [27]. In the program’s first formal evaluation, the “inclusion of both elements at the same time” was, especially in the beginning of the implementation phase, challenging for the service providers and confusing for the patients, causing some misunderstandings, frustrations, or even resistance to and non-acceptance of the new program [30]. 

During the program’s development phase (2018), four different isPO PIMs (leaflet, poster, patient information map, and website) were designed with a top-down approach, aiming to inform newly diagnosed cancer patients about this new program. The material was designed by the project leaders that included the German Cancer Society North-Rhine Westphalia (KG-NRW), which is responsible for the isPO care network support during program implementation. Both organizations possess a high level of content competence. Unfortunately, the isPO stakeholder “patient representative”, represented by the House of the Cancer Patient Support Associations of Germany (HKSH-BV), was only requested to provide general feedback to those materials after finalization. Before implementing the PIM in practice (2019), due to time constraints, no PIM quality assessment, end-user comprehensibility, and service-provider usability checks were conducted. Furthermore, no patient-information strategy was developed for a systematic assessment of the completeness of the PIM [31]. When conducting the external formative program evaluation, the evaluators were constantly engaged in different activities (e.g., observations, focus groups, and program quality workshops) with isPO stakeholders (e.g., service providers and network supporters). During the first formative evaluation that was conducted in the early program-implementation phase (June 2019), service providers of all four isPO networks claimed in individual interviews and focus groups that the initial isPO PIM urgently required optimization, e.g., in terms of quality and usability [30]. Furthermore, it turned out that PIM was utilized in a different manner or even partly ignored in the four care networks [30]. In the cross-sectoral isPO quality workshop (September 2019), in addition to the service providers, the German Cancer Society North-Rhine Westphalia (KG-NRW) in its role as network supporter, and the House of the Cancer Patient Support Associations of Germany (HKSH-BV), in its role as “active patient voice”, both emphasized that the currently utilized PIM was better suited to academic project information than as end-user program information. The before-mentioned stakeholders and the external evaluation team suspected that the insufficient quality of the PIM does negatively influence both end-users´ program and study acceptance. Hence, it was agreed by all participants of the workshop that the initial top-down designed four PIM should be assessed concerning their quality and subsequently optimized. In order to address the specific needs, the PIMs’ target groups (end-users and service providers), it was decided to actively involve them in this process. 

### 1.3. Aim

Works in the literature that describe, in detail, how to design, assess, and optimize PIM with patient participation are rare [18]. In health research in Germany, comprehensive patient involvement and engagement (PPI) with a high degree of participation is still rare or even considered as not appropriate (e.g., when conducting evaluations). However, formative evaluation should help minimize a new program’s teething problems by providing optimization suggestions [32]. By applying the critical-friend approach, the isPO evaluation team may serve as an impulse provider, provoking a look at the issue (e.g., PIM) with another perception and may foster development of a co-creative learning process [33]. 

The specific aim of this paper is to describe the bottom-up quality assessment and optimization process of the initial isPO PIM by applying the PHR approach with constant engagement of the patient representative. First, the evaluation team felt it is crucial to understand how the initial PIMs’ quality is perceived. Second, we aimed to involve the target group (cancer patients) in the PIM optimization process, as we were convinced that, due to their valuable contribution, the optimized PIM will turn out to be end-user friendly, high quality, and complete.

## 2. Materials and Methods

### 2.1. Optimization Approach 

Participatory health research (PHR), a bottom-up approach, was applied [34] as a supplement to the formative evaluation activities in order to optimize the initial isPO PIM. PHR’s leading principle is shifting power from the “experts” (e.g., professional researchers) to those stakeholders who possess insightful knowledge (e.g., patients) and/or experience (e.g., service providers) by enabling high participation [34]. Cornwall´s participation typology differentiates between six participation degrees: (1) co-option, (2) compliance, (3) consultation, (4) cooperation, (5) co-learning, and (6) collective action. It was utilized to describe the relationship between the researcher and stakeholder during the optimization process [35]. 

### 2.2. Roles and Competences

In order to gain a profound understanding of the quality requirements, a temporary PIM optimization team was formed, consisting of eight individuals with different backgrounds (Table 1), including patients´ representatives [36]. The patient representatives and the network support experts were constantly engaged as co-researchers with high participation degrees (degree 4/5). Moreover, respective end-users (exemplified by different German cancer survivors, representing their self-help group cohort) and isPO service providers (e.g., psychotherapists and isPO case managers) contributed with their valuable knowledge during the iterative optimization loops [37]. 

### 2.3. The PIM Optimization Process

**Figure 1:**
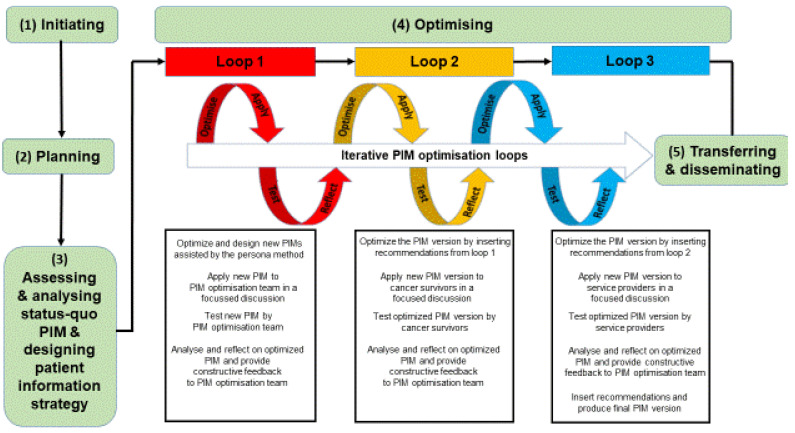
Optimization process of the isPO patient information material (PIM).

Our PIM optimization process contained five phases: (1) initiating, (2) planning, (3) assessing the status-quo, (4) optimizing, and (5) transferring and disseminating (Figure 1).

Initiation phase: The PIM optimization team was formed, the mutual goal was defined, responsibilities were negotiated, and it was agreed to conduct the optimization process bottom-up with the PHR approach (Figure 1, left side). 

**Figure 2:**
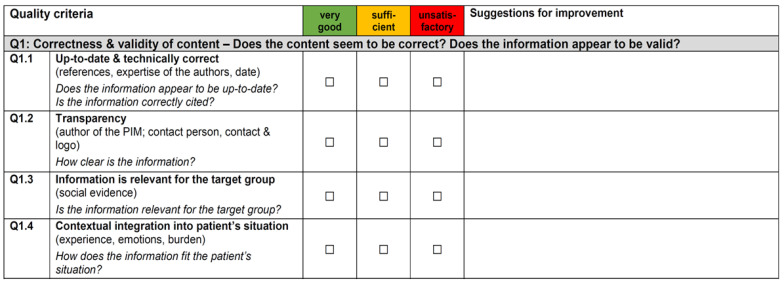
Extract of PIM assessment instrument UPIM-Check.

Planning phase: As no German appropriate end-user (patients)-friendly quality assessment instrument was found for our purpose, a new instrument, the User-friendly Patient Information Material Check list (UPIM-Check), was developed in a participatory manner and piloted (Figure 2). The UPIM-Check is divided into two parts: (1) assessment and (2) improvement. A traffic-light system (green = very good, orange = sufficient, and red = unsatisfactory) is used for the quality assessment. It consists of 31 criteria within four categories, namely correctness and validity of content, readability of content, structural readability, and graphical readability. Next, users may insert individual optimization impulses for each item in a free text field (see Figure 2). Details on the UPIM-Check’s development and validity are published elsewhere [38].

Moreover, the team discussed and agreed on the general assessment and optimization process, which included groups or numbers of participants, responsibilities, communication lines, resources, and data management. Finally, a plan of action was approved (Figure 1, left side).

Assessing and analyzing phase: First, the eight individual PIM optimization team members (Table 1) assessed the initial isPO PIM’s quality independently, using the UPIM-check (Figure 1, left side). Patients’ perspective was represented by the two members of the House of the Cancer Patient Support Associations of Germany (HKSH-BV). Next, eight isPO service providers (two from each of the four networks) assessed the material with the UPIM-check. Results were collected, merged into one overarching table, and distributed amongst the optimization team. Outcomes were thematically analyzed separately, distinguishing by PIM team, service providers, and end-users [39]. During two workshops (3 hours each), the optimization team conducted further analyses on the initial isPO PIM, distinguishing its strengths and weaknesses and summarizing the crucial points for improvement by focusing on its end-user friendliness and usability (Table 4 and Table A2) [17,20,40]. As the initial PIM was considered as being incomplete and unstructured in its utilization, the team developed a patient-information strategy.

Optimizing phase: Based on the PIM assessment’s findings, the iterative optimization process started (Figure 1, center). Participatory Action Research (PAR) in three overarching optimization loops was conducted [37]. Each loop contained 4 steps: optimize, apply, test, and reflect [41]. 

The first PAR loop was conducted by the PIM optimization team (Table 1) itself. First, two isPO PIM elements were improved, and two elements were newly designed, according to the patient information strategy (Phase 3), with the aim to adequately address the end-users’ (cancer patients and service providers) needs (optimize). For that purpose, the first author (belonging to the external impulse provider group) applied the persona method, resulting in an objective creation and design of potential isPO end-users types (adult cancer patients) [42]. Second, together with these personas, the new and optimized material was presented to the entire PIM optimization team (apply). They critically assessed the quality of the optimized PIM regarding its end-user friendliness and usability (test), and finally provided valuable recommendations for their improvement (reflect). Moreover, they reassessed its correctness, validity of content, and completeness according to the patient-information strategy. 

During the second PAR loop, the potential program´s end-users (cancer patients) were engaged during a three-hour focus-group discussion with a high degree of participation (co-learning, degree 5). Four cancer survivors, representing different cohorts of cancer self-help groups, and the federal consultant of the support associations (member of the PIM optimization team; see Table 1) participated. They concentrated on assessing and optimizing the end-user-friendly comprehensibility of content (e.g., understandability and terminology) and readability concerning structure and graphics (e.g., font, layout, and design) [7]. 

In the third loop, eight isPO service providers (e.g., case manager and psychotherapist) were engaged in a 90-minute focused discussion. The optimization concentrated on the usability of the PIM in the daily routine of the service providers (e.g., program information, and counseling) and its completeness.

Transferring and disseminating phase: In March 2020, the optimized and completed PIM was made accessible to the four isPO networks. Since then, this optimized PIM is utilized within all four isPO networks in the same manner and intensity for program information and orientation (Figure 2, right side). 

### 2.4. Ethical Considerations

During the entire optimization process, co-researchers´ (Table 1) and participants’ participation was on a voluntary basis; no incentives were offered. Ethical principles, such as mutual respect, equality and inclusion, democratic participation, active learning, and personal integrity, were considered during this PHR project [34]. All participants provided a written informed consent before data collection. 

## 3. Results

The bottom-up optimization of the isPO PIM lasted five months (09/2019–02/2020). Four PIM outputs that build on each other were achieved by considering high quality, user-friendliness, usability, and completeness. First, we present the results of the process outputs: (1) assessment of initial materials, (2) development of a patient information strategy, (3) optimization of initial materials, and (4) design of new PIM. Finally, a pre–post example of an optimized PIM is given.

### 3.1. Assessment of the Initial IsPO-PIM 

During the assessment phase of the initial isPO-PIM (Figure 2, phase 3), the entire PIM optimization team (Table 1) worked in a co-creative process together, shared their knowledge, and created a new understanding towards the required needs (participation degree five—co-learning) [35]. It became apparent that not all materials were utilized in each network by the isPO service providers for patient program information and orientation. Table 2 gives an overview of the four PIM elements and their imbalanced utilization. 

As only the leaflet and patient-information folder were universally utilized, in the following quality-assessment process, we focused on these two PIM elements. The initial isPO leaflet was assessed by 18 individuals with the UPIM-Check (Figure 2) from three perspectives: the PIM optimization team (*n* = 8), isPO service providers (*n* = 8), and end-users (*n* = 2), represented by cancer survivors. The initial isPO leaflet was assed as “unsatisfactory” in 11 subcriteria that were spread over all four quality criteria (Table 3, upper part). The key recommendations of the 18 individual assessments are summarized in Table 3, lower part). The assessment of the patient-information folder was conducted by the same participants (*N* = 18). Many redundancies among leaflet and information folder became visible, and weaknesses in all four quality categories were detected (see Table A1 for the recommendations given by the participants).

### 3.2. Development of the Patient Information Strategy

The isPO PIM’s assessment showed that the initial four PIM elements (Table 2) appeared to be incomplete, that information was partly redundant, and that its information provision was unsystematic and unstructured. Hence, the PHR team co-created an isPO patient-information strategy (Table 4) during the first PIM optimization workshop (see Figure 1, left side) whilst working in a co-learning atmosphere (participation degree 5) [35]. The strategy aimed to clarify (1) which isPO PIM components were essential, (2) the respective PIM’s target group, (3) the purpose, (4) the moment of utilization, and (5) the information specification. The co-creative process led to the decision that the complete isPO patient-information strategy should contain five PIM elements: a poster, a leaflet, a patient information folder, a one-pager (study information overview), and an end-user friendly website. 

The website was considered as important for augmenting the program´s transparency, dissemination, and to potentially increase the level of awareness beyond the current catchment areas. However, as designing an end-user-friendly isPO website (with several subpages) is a complex process that requires sufficient resources, it was decided that this work package needed to be separated from the other four elements. Thus, the website design’s experiences and outcomes are published elsewhere.

### 3.3. Optimizing the Initial IsPO PIM

When optimizing the initial PIM, experts by experience (cancer survivors representing their self-help peer group cohort) and isPO service providers contributed with their insightful knowledge and experiences (from participation degree four = cooperation to participation degree five = co-learning) [35]. 

The external impulse providers (see Table 1) facilitated and stimulated the entire PHR research process, structured and managed the three PAR loops, and inserted the optimization requirements in the different PIM elements. The three potential isPO end-users types (personas), created by the persona method [43], supported the external impulse providers in assessing the suitability of the optimized product in each loop. One isPO persona example is offered in Table A2.

**Figure 3:**
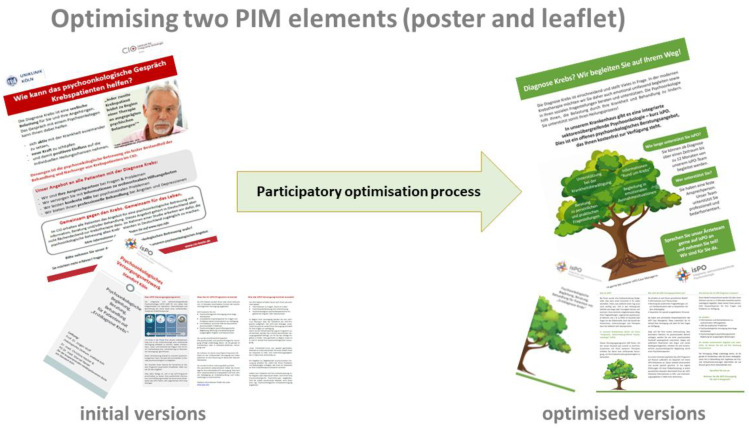
Example of the outcome optimization process on two elements of the isPO PIM.

Following the logic of the patient information strategy (Table 4), the leaflet builds on the information of the poster. Therefore, a corporate design was chosen to help patients recognize that both materials belong to the same program (e.g., a tree image with strong roots and an “encouraging” green for headings and subheadings). In its user-friendly wording and design, the new leaflet complements the poster and clearly addresses end-users’ needs (Table 4). It includes easy-to-understand elementary information about the isPO program and an empowering recommendation for action that is applicable in all four care networks. Easy-to-find contact details (e.g., case manager) are placed directly on the leaflet. As specifically recommended by cancer survivors in the assessment phase, Arial font size 12 was used to improve end-user-friendliness. The language was improved with the use of positive and resource-oriented words (e.g., “isPO can support you with your fight against cancer”) and completely avoiding technical terms. Finally, it was highlighted that isPO is “free of charge” and supportive for the biomedical cancer trajectory (Figure 3).

The optimization of the patient-information folder resulted in significantly fewer pages (from five to three pages). It appears as one document that personally addresses the end-user (Table 4 and Table A1). Its wording and layout were tailored (e.g., sentence structure and subheadings) to the end-users’ (cancer patients) needs. Redundancies and over-complex information regarding the contextual and legal frameworks of the isPO program (e.g., legislative information or paragraphs) were deleted. The benefit of participating in the isPO program and study was emphasized by using neutral wording. Furthermore, the point was stressed that isPO is offered to all newly diagnosed cancer patients, regardless of the degree of perceived emotional or social burden. The recommendation for action was formulated with empowering words and stands out graphically. 

### 3.4. Designing the New IsPO PIM

The design process of the new PIM was achieved in a co-creative manner (participation degree five, co-learning), as all PIM optimization team members shared their knowledge and experiences for this purpose [35]. Moreover, cancer survivors and isPO service providers also contributed as co-researchers. We designed two new components: the poster and a one-pager (see Table 4).

According to the patient information strategy (Table 4), the poster is the first PIM element that will inform about isPO in the care networks. Instead of the red “attention calling” color theme, an “encouraging” green was used (Figure 3). In the center of the poster, a tree image is placed as an eye-catcher and, simultaneously, as a reassuring and encouraging element. In the treetop the four general isPO care-support offers are formulated as possible actions. Furthermore, initial information on the stepped psycho-oncological care approach is provided. Considering the new corporate design, the poster includes a clear recommendation for action: “patients should ask their doctor in charge about isPO and should seek support”.

The one-pager aims to give an overview on the isPO study (Table 4). It was designed to transfer the compulsory study information (a twelve-page informed consent form) into a user-friendly format and language. It contains an easy-to-understand graphical representation of the isPO timeline (12 months) and shows the course of events within the isPO support program (e.g., intake) and its study (e.g., timing and type of data collection). Moreover, crucial points of the study consent form are summarized and highlighted, and references are provided to the in-depth information and its corresponding page reference in the informed-consent form.

After completing the third optimization loop (Figure 1), no further optimization suggestions were made by participants of the PAR process. Hence, the PIM optimization team was convinced that the four PIM elements (outputs) consider end-users’ (newly diagnosed cancer patients and isPO service providers) needs adequately. Therefore, the optimization process resulted in the availability of four end-user friendly isPO PIM (outcome). These PIM meets the quality criteria, as well as the specific needs of its end-users (e.g., correctness and validity of content, readability, and comprehensibility) and isPO service providers (e.g., usability). Figure 3 gives an example on the outputs pre–post. The new four PIM elements and patient information strategy were introduced to all care networks (network coordinator and head of psycho-oncological care) during the regularly occurring isPO quality workshop (February 2020). Since then, the optimized PIM have been similarly applied and utilized in the four care networks. During further external formative program evaluation rounds in 2020, including focus groups and interviews with end-users and service providers, it became evident that the optimized PIM positively assists service providers in their isPO care provision and helps newly diagnosed cancer patients to understand and accept the new program [43]. Their user-friendly approach turned out to be vital for the program’s stability, since, due to the Corona pandemic, in March 2020, face-to-face intakes were forbidden for some weeks. During this time, PIM were sent via e-Mail or letter before the intake. The feedback of the service providers and patients was very positive, especially for the leaflet and the one-pager [43]. 

## 4. Discussion 

This article reports on the experiences of optimizing the quality of PIM for the new complex German psycho-oncological care program isPO by applying participatory health research (PHR). Hereby, the patient perspective was constantly included in the process, as two members of the House of the Cancer Patient Support Association, representing the active voice of “experts by experience” [44], participated as co-researchers as an integral part of the PIM optimization team (Table 1). Furthermore, during the assessment and optimization process, six respective end-users (cancer survivors) and eight isPO service providers contributed with their knowledge with a high degree of participation (Figure 1; participation degree 4 = cooperation/participation degree 5 = co-learning) [35]. By involving PIM’s end-users, we made a very positive difference to the initial PIM development process, where material was developed by the project managers and experts for the program’s end-users, but without engaging them (participation degree 2 = compliance) [35].

We experienced that the PIM optimization team composition, containing patient representatives, experts, and researchers (Table 1), was crucial for gaining a multi-perspective comprehensive understanding of the real needs of the end-users, as also experienced by other researchers in different settings [21,45]. By incorporating both experiences and skills, we also felt that three issues were crucial: (1) to choose the team members carefully, (3) to constantly monitor the team composition, and (3) to be open for necessary adjustments [36]. Moreover, all team members (co-researchers and external impulse providers) benefited from the open, power sharing, and co-creative working atmosphere [46]. 

Before implementation, investing in a systematic PIM quality assessment seems to be imperative, as is also highlighted in other studies [16]. Even though patients possess a good understanding of their informational needs [47,48], only recently their perspectives got actively included in PIM quality assessments [17,47,49]. After reviewing the existing literature, the PIM team perceived the existing PIM assessment instruments as unpromising for end-users’ application, e.g., by not considering end-user’s health-literacy level. To the best of our knowledge, previously “no suitable” end-user friendly quality assessment instrument existed in Germany. We developed and tested our UPIM-check instrument in a co-creative manner with a strong contribution from the patient representatives [39]. In this context, we declared *suitability* according to four of the six categories defined as follows [20]: content, graphics, layout/typography, and learning stimulation and motivation for the decision-making process. After the UPIM-check utilization, the involved experts, researchers, service providers, and end-users perceived the instrument as “suitable”, “surprisingly end-user friendly” and “resource saving in its application”. End-users (cancer patients and service providers), in particular, welcomed the UPIM-check as the instrument empowered them to actively participate in the scientific work. This, in turn, prepares them in the long term to contribute to the optimization processes on its highest level of participation degree 6 = collective action [35]. UPIM-Check turned out to be helpful to identify the shortcomings and limitations of the initial isPO PIM from three perspectives: experts, patients, and service providers. The need for gathering a multi-perspective understanding was also considered as important by other researchers [50]. As suggestions for its optimization were offered by the three different groups, we were confident to address several important quality criteria when preparing the material for the first PAR loop. Regardless, it still needed three loops until we were confident that the maturity of all PIM (high quality and user-friendly) was fully achieved. During further formative evaluations (2021), it became evident that optimized material helped potential end-users to better comprehend the new program, leading to better program acceptance [43]. Moreover, service providers highlighted that, especially the “one pager (Table 4), was very helpful for informing newly diagnosed cancer patients about the program. Even with the Corona pandemic, the PIM turned out to be appropriate for the program orientation and intake [43]. If the program is rolled-out to nationwide care, it might be helpful to invest in an APP, e.g., as a platform for PIM. However, developing an APP is a complex process itself that needs sufficient resources (e.g., time, design thinking, financing, or knowledge). Moreover, in the development of an end-user-friendly APP, we recommend the constant engagement of end-users. 

Due to time constraints, in the beginning of the isPO program implementation, the importance of a so-called “patient information strategy” was undervalued. However, after investing sufficient resources into its development, it helped to define, structure, navigate, and complete the analogue PIM for our new and complex program. Due to the different informational needs, we propose to develop such a strategy as part of the implementation strategy, as also suggested by Huynh et al. [51]. 

### 4.1. Designing and Optimizing High-Quality PIM Bottom-Up 

In Germany, PHR, patient involvement and engagement in research is still rare [52,53,54]. By engaging co-researchers early, proactively, and with high participation degrees ^23^ during the PIM optimization and designing process, an innovative shift in power towards the co-researchers occurred. Our PIM benefited from these conditions, as also promoted or experienced by other researchers [18,21]. Additionally, we experienced that the criteria correctness and validity of content, readability in respect to text structure and graphics, comprehensibility for the end-users, and their utility in practice were crucial to achieving the high quality of our PIM. However, we believe that these criteria can only be accomplished if a program’s end-users (e.g., cancer patients or service providers) are invited to participate in the PIM design or optimization process. In our case, end-users´ recommendations had a high impact on the readability and comprehensibility, whereas the feedback of the service providers reflected on the utility, as also highlighted by Cook and colleagues [55]. We are certain that the optimized PIM addresses the needs of the program’s end-users, because it underwent serval pilot loops via PAR, as recommended [37,55], before its dissemination to the field. By prioritizing high participation with so-called experts by experience (e.g., farmer cancer patients), we perceived PHR also as bridging the gap between theory and practice [47,56]. 

We demonstrated that designing a high-quality PIM requires time, resources, in-depth contextual understanding, and an appropriate approach [3,21,57]. The effect of a detailed understanding of end-users’ needs (e.g., comprehensibility) on the program´s acceptance by those end-users was underestimated by the program designers when developing the PIM top-down [50]. We chose PHR, a bottom-up approach, as it is perceived as a strategy to overcome the gap between academic researchers or experts and end-users (e.g., patients) in practice [23,46]. Hereby, both the co-creation of new practical-based knowledge and the empowerment of co-researchers was achieved by a high degree of participation [34,35,58]. Overall, our entire PIM optimization team (Table 1) and the different participants of the study experienced the high degree of participation as empowering. However, they varied in regard to their specific role and earlier engagement experiences between “advantageous and practicable” (experts), “expedient and impulse giving” (academic researchers), “innovative and empowering” (isPO service providers), and “democratic and highly welcoming” (patient representatives) [30]. 

### 4.2. Strengths and limitations

Optimizing PIM with the PHR approach requires the availability of resources (e.g., time, skills, and staff) and an appropriate access to the end-user group [59,60]. In our case, both were available. PHR requires constant openness for power sharing and respect for accepting democratic rules from all participants [34]. Seven of the eight members of the PIM-optimization team were novices in applying PHR. We experienced that, despite the increased attention for participatory approaches, in the German health-research domain, the benefit of participative approaches remains largely undiscovered. The lack of experience of most members of the team required intense methodological guidance in order to hold the team on the “PHR road”. Hereby, the methodological experience of the external impulse provider as a facilitator was imperative. The fact that all participants were committed to the PHR approach was also helpful. To set clear mutual expectations and agree on conflict-resolution mechanisms right in the beginning of a PHR process seemed to be crucial [36]. Overall, the PIM´s optimization with the PHR approach was perceived as communication intensive, as also experienced by others [61]. It required strong communication and mediation skills from the facilitator (external impulse provider, first author). However, investing constantly in knowledge transfer and skills training resulted in co-researchers´ empowerment [61]. Finally, it was more time-consuming than expected (2 months longer). We learned that, beforehand, sufficient resources (e.g., time and staff) should be calculated for PIM [36,61]. 

New context-specific bottom-up knowledge was gathered from multiple perspectives (e.g., patients, isPO service providers, and experts). Therefore, we are certain that PIM should fit the real needs of service providers (e.g., utility) and patients (e.g., understandability) in practice [18,21]. However, the optimized PIM is limited to (1) the geographic coverage of our program, (2) end-users (cancer patients) needs, (3) German reading skills, and (4) a moderate health-literacy level. Therefore, a generalization of the optimized PIM is restricted.

The optimization process was started by assessing the initially used isPO-PIM. Our instrument (UPIM-Check) that was developed for this purpose was perceived as a “highly valuable instrument”. End-users, isPO service providers, and the PIM optimization team experienced it as user-friendly during assessment and resource saving by generating recommendations for optimizations from the same perspective. Currently, this instrument is validated and accessible free of charge to the German-speaking society only [38,62]. However, an English version is currently being piloted with self-help organizations in the UK, USA, Canada, and Australia. 

## 5. Conclusions

The optimized PIM facilitates isPO service providers’ care provision and helps newly diagnosed cancer patients in understanding and accepting the new psycho-oncological care program (isPO). The correctness and validity of content, readability, comprehensibility, and utility of the PIM fit to end-users’ needs and, therefore, simplify isPO service providers’ work.

PIM optimization benefited from applying PHR, besides the fact that the bottom-up approach required resources. The patient representatives’ contribution and active patient engagement were central for quality assessment and designing needs-driven, mature, and complete PIM. 

Critical reflection on research and power sharing stimulated the entire PIM optimization team. Finally, both the PIM team and co-researchers were empowered through the mutual learning process that may positively impact further program implementation. 

## Figures and Tables

**Table 1:** Composition of the isPO PIM optimization team.

Affiliation and Number of Participants	Role during the PIM Optimization Process	Role in IsPO	Overall Expertise
House of the Cancer Patient Support Associations of Germany (HKSH-BV) 2 participants	Co-researcher patient perspective Assess PIMs’ quality Articulate new cancer patients’ real informational needs Provide optimization impulses	Accompany and advise program designers Recruiting and training volunteers to be part of the isPO care program	Uniting different cancer self-help groups Longstanding experience in representing patients´ perspectives and requirements on different societal and health political committees at national and local level Experts by experience
German Cancer Society North-Rhine Westphalia (KG-NRW) 3 participants	Co-researcher expert perspective Assess PIM quality Actively support the optimization of the initial PIM and co-creation of new PIM Explore PIMs’ usability in the new care networks	Support the four isPO care networks Were partially engaged in the design of the initial PIMs during the program’s development phase	Experts for psycho-oncological care structures Multi-professional background in health science Profound experience in improving structures for regional cancer support
Institute for Medical Sociology, Health Services Research and Rehabilitation Science (IMVR), University of Cologne 3 participants	External impulse provider (critical friend approach) Facilitating and stimulating the PHR research process Contributing scientific, technical and managerial knowledge, Assuring a constant communication flow and data storage	External evaluation of the isPO program	Academic researchers with expertise in public health, health services research, sociology, and psychology Experience in health system development and evaluation

**Table 2:** Utilization of the initial isPO-PIM in the four care networks.

Initial IsPO PIM Elements	Care Network 1	Care Network 2	Care Network 3	Care Network 4
Leaflet	X	X	X	X
Patient information folder	X	X	X	X
Poster	X			
Website of the care network (program subpage)	X			

**Table 3:** Multi-perspective outcomes of the quality-assessment process of the initial program leaflet.

**Leaflet Weaknesses Listed by the Four Quality Criteria of the UPIM-Check (Part 1 UPIM-Check)**				
**Quality Criteria**	**Criteria that Were Assessed with Red (=Unsatisfactory) within the UPIM-Check**	PIM Optimization Team *	Service Providers *	**End-Users** (Cancer Survivors) *
Correctness and validity of content	Contextual integration into patient´s situation	8		2
	Relevance of the information	6		2
	Recommendation for action	8		2
	Motivation and increase of self-efficiency	8		2
Readability of content	Aim for the patient identifiable	6	8	2
	Simple, clear language	8	8	2
	Use of empowering words	8		2
Structural readability	Appropriate sentence complexity	8	8	2
Graphically readability	Layout/overall visual appearance	7		2
	Appealing “eye catcher” functioning as a “door opener” for recruitment	8	8	
	Illustrations	7		2
Key Optimization Recommendations (Part 2 of UPIM-Check)				
**Perspective**	**Summary of Key Optimization Recommendations**			
PIM optimization team	isPO must be “easy to distinguish” and to differentiate from other support offers (e.g., sport and music therapy); PIM should contain both “catchy information” and “straightforward positively phrased recommendations for action”; Adhere to the “taxi principle” (pick up the patient in his/her current situation) to sensibly consider patients’ emotional state and health literacy after receiving a life-threatening diagnosis Complete PIM by adding information on isPO that support: (1) is offered to newly diagnosed cancer patients, (2) according to their individual needs, and (3) that no specific number of sessions “must” be attended within the 12 months.			
isPO service providers (psychotherapist, case manager)	Illustrate the isPO trajectory, e.g., by using a timeline; Highlight that isPO is a “free of charge” program; Diminish the variety of terms or technical terms; Improve the language by using a positive and resource-oriented words and by avoiding negative terms such as “fear” and “depression”.			
End-users (cancer survivors)	Apply a patient-friendly language (e.g., shorter and better structured sentences); Pay attention to the utilization of different terms (e.g., instead of using words such as “project”, “study”, or “concept”, the word “program” should be constantly utilized); Utilize positive and empowering words; Apply a comforting und reassuring design; Choose a suitable font and font size (e.g., Arial, 12 pt).			

* Number of assessments by the respective groups (PIM optimization team, service providers, end-users).

**Table 4:** Patient-information strategy applied to PIM elements and hierarchical order.

PIM Elements (o or n) *	Target Group	Purpose	Moment of Utilization	Information Specification
Poster ^n^	All patients	Display/present the existence and purpose of isPO (“door opener”, motivator)	Broad (waiting room area, general hospital area)	General information concerning isPO Focus on available support and resources
Leaflet ^o^	Potential isPO-patients	Specific (first introduction to isPO, multiplication factor)	Soon after the first cancer diagnosis	isPO-specific and end-user oriented information (clarification of the benefits) Relevant elements:
				Contact person Recommendation for action
Patient information folder ^o^	Suitable isPO patients	Briefing the patient	During the introductory conversation (intake)	Crucial isPO program and study details
One-pager ^n^	Patients that should be enrolled in the isPO study	Enrolment (to provide a comprehensible overview regarding the study and all ethical aspects and informed consent)	Enrolment	Overview and orientation (e.g., reference to pages in the ethical consideration paper)
Website ^n^	All patients and other interested persons (e.g., researchers)	Broad (to increase motivation, to raise awareness for psycho-oncology and isPO)	Various (when individually needed for patient information during research)	isPO-specific and needs driven information

Note: * ^o^ = optimized PIM, ^n^ = newly designed PIM.

## Data Availability

The data are not publicly available due to ethical and legal restrictions, as participants of this study did not agree for their data to be shared publicly. Upon reasonable request, the data presented in this study are available from the corresponding author.

## Appendix A

**Table A1:** Multi-perspective recommendations for optimizations of the initial program patient information folder.

Patient Information Folder: Key Optimization Recommendations (Part 2 of UPIM-Check)	
Perspective	Summary of Key Optimization Recommendations
**isPO service providers** (psychotherapist and case manager)	Utilize an “empowering and welcoming” recommendation for action; Clarify exactly “what patients personally need to do in order to join isPO”.
**End-users** (Cancer survivors)	Clarify that isPO is a “free of charge” program; Provide transparent information and keep the necessary information “short and concise”; Delete over-complex information regarding contextual and legal frameworks of the isPO program (e.g., between the lead institution and health insurers and legislative information or paragraphs); Emphasize that isPO is offered to all newly diagnosed cancer patients, regardless of the degree of perceived emotional or social burden.

**Table A2:** Persona example developed to support the isPO PIM optimization.

**Target Group: middle-aged man** **Individual Characteristics:**		
Is still fully in professional life and would like to realize himself there; Life motto: “You can do anything if you make an effort”; Initial diagnosis of prostate CA.		
***Name:*** Albert	***Age:*** 56 Years	***Family status:*** married, 3 adult children
***Gender:*** masculine	***Occupation:*** Head of car dealership, car mechanic (master)	***Education level:*** secondary school leaving certificate
***Living environment:*** Lives contentedly with his partner in a medium-sized city; Rental apartment on the outskirts; good neighborhood relationship; Children have already moved out and live in southern Germany (350 km distance).		
***Interests and habits:*** Loves his work in the dealership and customer contact; Likes to read (newspapers, crime novels); Often goes on short bike tours with friends at the weekend; Plays amateur football with the old men; Once-a-year vacation in Mallorca with his wife.		
***Personal goals:*** Exercise your job for as long as possible; Be a good partner and responsible father.		
***Frustration and Limitation due to cancer diagnosis:*** Fear that everything will change as a result of the cancer diagnosis (e.g., not being able to cope with everyday life, becoming dependent, not being able to perform professionally); Fear of becoming impotent; Being afraid of dying; Fear of pain.		
